# The acceptability of conducting data linkage research without obtaining consent: lay people’s views and justifications

**DOI:** 10.1186/s12910-015-0070-4

**Published:** 2015-11-17

**Authors:** Vicki Xafis

**Affiliations:** Discipline of Public Health, The University of Adelaide, Adelaide, Australia; Discipline of Paediatrics, The University of Adelaide, Adelaide, Australia; Sydney Children’s Hospitals Network, Sydney, Australia

## Abstract

**Background:**

A key ethical issue arising in data linkage research relates to consent requirements. Patients’ consent preferences in the context of health research have been explored but their consent preferences regarding data linkage specifically have been under-explored. In addition, the views on data linkage are often those of patient groups. As a result, little is known about lay people’s views and their preferences about consent requirements in the context of data linkage. This study explores lay people’s views and justifications regarding the acceptability of conducting data linkage research without obtaining consent.

**Methods:**

A qualitative study explored lay people’s views regarding consent requirements in data linkage via four hypothetical data linkage scenarios of increasing complexity. Prior to considering the scenarios, participants were provided with information regarding best practice data linkage processes via discussion and a diagrammatic representation of the process.

**Results:**

Lay people were able to understand the intricate processes involved in data linkage and the key protections afforded within a short amount of time. They were supportive of data linkage research and, on the whole, believed it should be conducted without consent provided a data linkage organization de-identifies the data used so that researchers do not handle identifiable data. Many thought that de-identified data holds a different status to identifiable data and should be used without specific consent in research that aims to benefit society. In weighing up conflicting values and interests, participants shifted consent preferences before arriving at their final consent preference for each scenario and provided justifications for their choices. They considered the protection of people’s information, societal benefits, and the nature and constraints of research and recognized that these need to be balanced.

**Conclusions:**

With some exposure to the features of data linkage, lay people have the capacity to understand the processes sufficiently in order to consider ethical issues associated with consent preferences. Shifts in views reveal the complexity of such decisions. While privacy protection remained an important consideration for most participants, adequate protection measures adopted in best practice data linkage were viewed by most as protection enough for data linkage to proceed without specific individual consent.

## Background

All interactions with the health care system generate individual files containing both personal and health information. Typically, collections of such data are used to facilitate the provision of healthcare and to enable the efficient and effective operation of healthcare systems. Beyond such uses of the data, however, combining these administrative electronic collections, e.g. medical records and deaths data, into single de-identified data sets provides researchers and policy makers with rich data of potentially great medical, epidemiological, economic, research design, and policy significance [1–3] even if such data sets present certain limitations for researchers, as a result of the primary purpose for which they were collected [4].

The key feature of best-practice data linkage processes involves separation of the various tasks required to achieve the linkage. This results in no single third party entity ever having access to the fully identifiable dataset that each data custodian holds. In addition to this key feature of data linkage, there are stringent site-specific processes such as technical and physical protections of data. There are also legislative requirements relating to the protection of information and researchers requesting the use of certain government datasets in Australia must provide a security plan outlining their organizational data protection requirements in addition to data protection methods researchers themselves devise to manage potential risks that might arise in data linkage activities [5].

One of the key ethical issues arising in data linkage research relates to consent requirements. Much has been written about patients’ consent preferences in the context of health research [6–16]. However, when these studies consider consent requirements in the context of data linkage [6, 10–12], data linkage research sometimes appears as one of many scenarios presented in the general health research context and has not been focused on exclusively. In addition, the views on data linkage are often those expressed by patient groups. As a result, little is known about lay people’s views and their preferences about consent requirements in the context of data linkage [17].

This paper considers lay people’s views on the acceptability of conducting data linkage research without obtaining consent via their consideration of four hypothetical scenarios. The findings demonstrate the complexity of the decision-making process when people have the time and requisite understanding to revise their consent preferences. Importantly, the paper also presents the reasons that support participants’ initial and revised views on consent preferences in data linkage. This provides insight into people’s underlying values.

The qualitative study reported on in this paper was a component of doctoral research forming part of a larger multi-disciplinary project titled *Vaccine Assessment Using Linked Data (VALiD)*. The study received ethics approval from the Women’s and Children’s Health Network Human Research Ethics Committee, Adelaide, as participants were drawn from a pool of parents whose babies had been born at the above hospital and who had participated in the VALiD Randomised Controlled Trial (RCT) [18], another component of the VALiD study. The inclusion criteria for the RCT were mothers with a live or surviving birth at the hospital selected, 18 years of age or older, and residents of South Australia. Excluded were mothers whose child had died, whose child had been in neonatal intensive care for two or more weeks, mothers who were incarcerated, mentally incapacitated, or whose child had been adopted or placed in foster care [18].

### Current knowledge about people’s consent preferences

Research on people’s consent preferences reveals that consent choices are not straightforward for those engaged in making them [7–16]. Some of the studies conducted highlight the value that people place both on the conduct of research and the protection of privacy while at the same time acknowledging the constraints that consent requirements place on research [12, 14]. Studies also show that lay people are able to discriminate between uses of identifiable and de-identified information [19].

A minority of studies showed evidence of a strong preference for consent for any research conducted [11]^1^, [16, 20]. Overall, however, findings seem to support the non-consensual use of data more so than the requirement for consent [8–10, 13, 21]. However, such conclusions are by no means straightforward and clear-cut. For example, when participants indicate support for non-consensual use of personal and health information, this is often done under certain specific and unique circumstances. An example of such considerations is participants’ views on the use of Veterans Affairs data where trust in this organization played an important role in the views expressed [14]. Moreover, participants’ responses regarding the acceptability of non-consensual uses of data vary considerably depending on clarifications regarding safeguards [21]. Furthermore, when the no-consent option is preferred, it is sometimes qualified with a preference for some form of notification or information provision about uses to which data are put [11, 14] or the ability to opt-out [6].

In some studies, there was much stronger support for the provision of consent in all research or studies related to the research described. This preference, once again, is not straightforward. For example, the kind of information being linked (even if de-identified) influenced participants’ views, with linkage of health information to information drawn from a variety of sources outside the health sector posing a concern [11]. In two studies consent was the preferred option but both consent options incorporated future use of data without the need to re-consent participants [12, 13]. Such responses may relate to issues of trust; that is, once participants have engaged with a group of researchers and trust them, they are willing for the information to be re-used by the same research team [20]. Unexpectedly, it appears that a lack of trust in de-identification processes relating to data linkage did not increase some participants’ desire to have greater control over their information via consent processes [10].

The literature indicates that people generally prefer to have some knowledge of the use to which their information is put, with a desire for greater or lesser degrees of information provision and control over the process. Few studies, however, have focused exclusively on lay people’s views or on the decision-making process and the shifts people make, when afforded the opportunity, and no studies have provided detailed justifications for people’s consent preferences. Such insights are only discoverable through qualitative research which can also clarify with further probing whether their choices are in fact what they intended.

## Methods

### Influences on research design

A number of assumptions were made at the outset: 1. Participants would generally have a limited understanding of what data linkage involves; 2. Most participants would want to opt in and provide specific consent; and 3. Participants would generally prefer to provide consent for both identifiable and de-identified data. Assumptions 2 and 3 led to the design of hypothetical data linkage scenarios, as described below, with progressive probing to uncover commitment to their initially stated consent preferences and their underlying values.

The assumptions were based on a preliminary general reading of some of the literature [11–13, 16, 20], the researcher’s extensive professional expertise in research ethics, and the general requirement for consent to be provided in all interactions concerning personal and health data.

### Research design

A small pilot study preceded the main study to determine if the scenarios were understood in the same manner by all, if there were any aspects which required amendment, and if the recruitment procedures were appropriate. The pilot study helped refine the research rules pertaining to recruitment methods but the interview schedule itself, including the scenarios, were not amended based on feedback received from participants.

### Sampling method

**Fig. 1:**
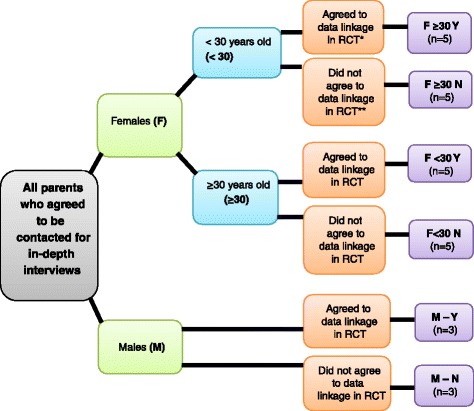
Purposive sampling method for identification of six pertinent groups. * “Agreed” means the parent returned a study form in the opt-in arm OR did not return a study form in the opt-out arm, thereby facilitating the data linkage of the child’s vaccination data to his/her hospital data Coded as Y. ** “Did not agree” means the parent returned a study form in the opt-out arm OR did not return a study form in the opt-in arm, thereby precluding the data linkage of the child’s vaccination data to his/her hospital data Coded as N

A stratified purposive sample (Fig. 1) comprising 20 females and six males was selected from a pool of 763 participants who had agreed to be contacted. Even though this was a qualitative study, the selection process adopted resembled that of a quantitative study. The sample was stratified to provide a systematic approach to selection given the large number of potential participants and to capture a variety of views (see Table 1). Of the 763 who had agreed to be contacted, 704 were females and 59 males. This is why fewer males were recruited and why males were not stratified by age. No exclusion criteria applied, other than not having been involved in the VALiD RCT. Participants were allocated to one of six groups based on gender, age (for women), and whether they had agreed or not agreed to data linkage in the RCT. Age (for women) was considered to be a feature that might provide variation in views, as young mothers, for example, are known to share different characteristics from those of older mothers [22].

**Table 1:** Participant characteristics

Pseudonym	Age	Level of education	Continent Born
Margaret	27	Tertiary	Australia
Mary	28	TAFE^a^ (incl. trades)	Australia
Molly	28	TAFE (incl. trades)	Australia
Mel	29	Tertiary	S Asia
Mandy	29.6^b^	Tertiary	S Asia
Helen	26	Yr 11 or equivalent	Australia
Holly	24	Yr 12 or equivalent	Australia
Haley	29	TAFE (incl. trades)	Australia
Henrietta	24	Tertiary	N America
Harmony	27	Tertiary	Australia
Teresa	41	Tertiary	Australia
Tina	39	TAFE (incl. trades)	Australia
Tracey	30	Tertiary	S Asia
Tegan	31	Tertiary	Australia
Trixie	39	Tertiary	Europe
Vanessa	35	Tertiary	Australia
Vallery	30	TAFE (incl. trades)	Australia
Victoria	32	Post graduate degree	Australia
Verity	33	Tertiary	Australia
Virginia	34	Tertiary	Australia
John	36	TAFE (incl. trades)	Africa
Jack	30	Tertiary	Australia
Jacob	31	Tertiary	S E Asia
Darren	25	Yr 10 or equivalent	Greater Middle East
Danny	31	Tertiary	Asia
Don	31	Tertiary	Greater Middle East

^a^Technical and Further Education institutions providing vocational tertiary education courses

^b^There was an error in the original data, which was only identified after the analysis of the current data. As a result, this participant was 29 and 6 months but was included in the 30+ group

### Recruitment

**Fig. 2:**
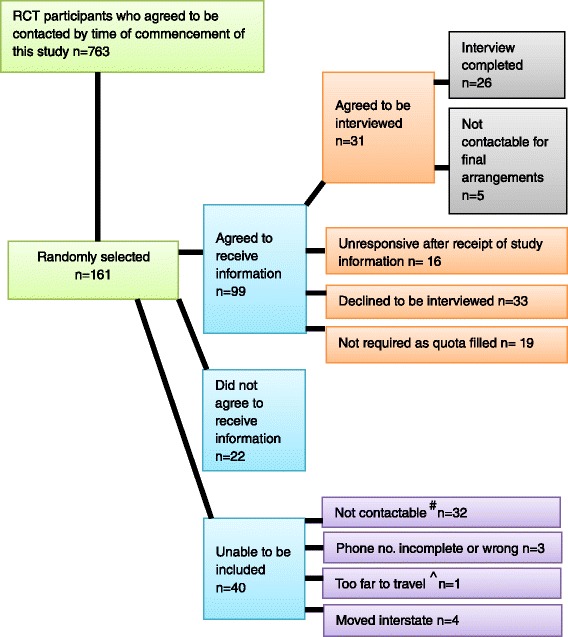
Interview selection figures. ^#^ Not contactable refers to calls not being answered, phones being disconnected, or unresponsiveness to messages left. ^^^The furthest travelled for an interview was 512 km by plane. It was later decided that such travel distances were not possible

Of the 763 individuals who had agreed to be contacted for this study, a total of 161 was selected on a rolling basis (Fig. 2). An initial phone call determined potential participants’ willingness to receive information about the study. In total, 476 calls were made to recruit 26 participants.

### Data collection

In-depth semi structured face-to-face interviews ranging from 45 to 90 min were conducted in participants’ homes. Interviews were audio taped and transcribed verbatim by a professional transcribing agency. Pseudonyms replaced all participant names.

### Interview schedule and conduct of interview

Prior to discussing the scenarios, participants were asked to describe what data linkage is. Following this, a best practice data linkage process was discussed with the aid of a diagram which participants could refer to at any stage of the discussion. Four scenarios, forming the main component of the interview, were discussed and participants were asked about their consent preferences for each scenario. ‘Consent preferences’ referred to the consent options: consent, no consent, or notification of use of data but no full consent process. Each scenario related to a hypothetical data linkage research project (Table 2). The scenarios displayed incremental complexity, i.e. Scenarios 1 and 2 involved only health data linked by experts, Scenario 3 involved health and criminal records data linked by experts (and consent was not given as an option), Scenario 4 involved health data, WorkCover records, and employment data to be linked by researchers (hence researchers would initially have access to identifiable data). As a result of the content and order of presentation, the scenarios also performed an educational function: Scenario 1 provided the *training*, Scenarios 2 and 3 enabled *application* of the data linkage and ethical considerations and Scenario 4 functioned as *testing* of participants’ understanding of concepts, as well as potential biases arising from participants’ willingness to provide responses they felt might be welcomed.

**Table 2:** Scenarios

Scenario 1- health data linked by experts
A study is being conducted by university researchers and they want hospital information (which includes the medical history, name, age, ethnicity, and postcode), a cancer register, and a deaths register to be linked with each other. The researchers want to find out if there is a link between lung cancer and living next to busy main roads. The findings will contribute to better town planning. In order for the study to be successful and so that it provides accurate findings to ultimately help with the management of some forms of cancer, it’s very important for everyone on the cancer register (several thousand people) to be included in the study. The researchers will never have any identifying information because they will not do the linkage themselves and all information that identifies people will be removed before they get the linked data.
Scenario 2 - health data linked by experts
Researchers in collaboration with the ambulance service are conducting research into cardiac arrests and resuscitation to see if call-out response times affect survival rates. They will need to have data about approximately 300,000 people on the ambulance databases, hospital admissions, and death registers linked. The researchers will never have any identifying information because they will not do the linkage themselves and all information that identifies people will be removed before they get the linked data.
Scenario 3 - health & criminal records data linked by experts
University researchers and researchers from a mental health organisation want to study violent behaviour in people experiencing mental health issues. They need to link about 50,000 mental health hospital records Australia-wide (including admissions and discharge information) with police incident information, such as calls for domestic violence. The researchers will never have any identifying information because they will not do the linkage themselves and all information that identifies people will be removed before they get the linked data. The Police will not have access to the mental health hospital records. Now still talking about the same research, the process of linking such a lot of information from a number of States/Territories is very complex, expensive and very time-consuming. So in this research, once linked, the identifying information will be removed but a key will be held separately. The key connects the identifying information of all the people on the databases and the codes they were given. The key will make it possible for researchers from various States/Territories to ask for de-identified information about their State/Territory so that they can do further research without having to have all the information linked from the start.
Scenario 4 – health, Work Cover & employment data linked by researchers
University researchers are conducting research on work-related stress on behalf of Work Cover to discover whether there is a link between increased levels of stress and work insecurity, for example caused by casual employment. They need to link 100,000 work stress claims containing identifiable general and mental health information with employment data including employment history, leave information, seniority level etc. for the same individuals who are employed at the Government organisations involved in the research. In this instance, the linkage will not be done by an independent team of data linkage experts. Instead, the researchers will do the linkage themselves. A report with de-identified findings will be made available to Work Cover. [In discussions with participants, it was made clear that work stress claims would be accessed from WorkCover^a^.]

^a^WorkCover is a Government agency aimed at preventing, compensating, and rehabilitating workers involved in occupational accidents or affected by diseases resulting from their workplace. See http://www.workcover.com/

The inclusion of data sets unrelated to health was deemed important, as this provided a contrasting effect bringing to the fore issues people considered pertinent when considering linkage of health data alone, compared to health and other data. If participants indicated that consent was required, a series of considerations, such as time and monetary constraints in the research context, integrity of the research as a result of low participation rates, and security of information were put to participants to see if their view regarding the need for consent would be affected. Following the discussion of individual scenarios, participants were also asked a more general question relating to the acceptability of using health data without consent for research purposes.

### Data analysis

Participants’ views and justifications for consent were analyzed using the *framework approach* [23–25], as it better tracks the processes adopted from raw data analyses to the interpretation of data therefore displaying greater transparency in the analysis of the data and the researcher’s interpretation of the data [25]. Numerous codes arose directly from the questions posed to research participants in relation to consent options. In some scenarios, however, additional codes arose from participants’ contributions. Codes were cross checked by an experienced qualitative researcher, Dr Victoria Wade. QSR International’s NVivo 8 software [26] was used to assist with the management of the data.

### Charts

A key feature of the Framework Approach is the use of charts, created by studying the raw data and summarizing them, which are fundamental to analyzing the data and are separate to the Tables provided in this paper. The charts developed provided a summary of participant consent preferences and justifications for each scenario. The themes identified in relation to justifications for their choices clustered around issues participants felt were important in each scenario. Some of these issues were stated explicitly and some were inferred by their responses.

The charts do not have pre-structured formats and are created in accordance with the data collected and the needs of the individual research project analysis. The charts developed in this study provided an innovative approach to recording people’s views and justifications which has not previously been employed. They display key information relating, not only to initial individual views, but also, importantly, to shifts in participants’ views, as well as the direction of such shifts. Tracking participants’ shifts in views provided an understanding of the manner in which these individuals arrived at their final decisions, the reasons why their views changed, and the complexity of the process involved.

## Results

### Participants

Participant ages ranged from 24 to 41 years and the level of education varied from year 10 to postdoctoral tertiary education (Table 1). The country of birth was not recorded to preserve greater anonymity but the general geographic region was recorded to demonstrate the variety of cultural backgrounds included in this group.

### Findings in relation to pre-research assumptions

Three assumptions were articulated before the conduct of the research. Assumption 1 was found to be true but Assumptions 2 and 3 were found not to hold.

#### Assumption 1: Participants will generally have a limited understanding of what data linkage is and what it involves

Participants generally did not remember the information provided as part of their involvement in the VALiD RCT that preceded this study. Their understanding of data linkage in many cases related to the misconception that data linkage is the sharing of personal and health information within the health care system. An understanding of the real nature of data linkage and its processes developed out of the explanation given during the face-to-face interviews with the assistance of the diagram used and referred to in the interview where required.

#### Assumption 2: Most participants will want to opt in and provide specific consent

The initial assumption proved to be far removed from what these lay people considered appropriate. Most participants considered it appropriate to conduct data linkage projects without consent provided that there is separation of tasks so that researchers do not obtain identifiable data. Participant quotes below highlight this point.

*But at the end of the day researchers aren’t going to have your personal information so it shouldn’t really matter*. (Vallery)

*So I just think that once the information is in there and it’s all numbers and letters, then it’s not an issue, I don’t think, no* (Teresa)

*If it's no information as in names and phone numbers and addresses of people is going to be let out then it should take place. It’s probably not that important to get the okay because it is just information* (Mary)

The responses provided in relation to the non-consensual use of health information matched the responses in relation to the scenarios, as the majority of participants indicated that consent was not required in data linkage projects because of the protections provided and the fact that data are de-identified.

Most participants preferred the no consent option rather than notification of the intent to use data, which at least alerts people to their inclusion in future research, both for specific projects and more generally for unspecified future uses.

#### Assumption 3: Most participants will prefer to provide consent for both identifiable and de-identified data

On balance, most participants believed that consent was not required for data linkage research. Many participants were clear that de-identified data should not be treated in the same way as identifiable data, as they believed that the fact that data could not be traced back to specific persons was morally significant. Many of the participants with this view also expressed the view that once the identifiers were removed, the information became completely detached from individuals and was just ‘information’ which could be used to benefit others in society. The quotes below illustrate this point.

*They’re just getting information. It’s not anybody. It’s A or B or a number, it’s not actually a person…. There is a separation from the person. So it’s just information, it’s not a person; it’s not a name or a phone number or an age. It’s just information*. (Mary)

*Yeah, because obviously the identity is not revealed. Supposing I’m number 24, even I won’t know that I’m number 24. So it really doesn’t raise a question about me not giving consent because you’ll just have in your chart that number 24 is whatever, whatever the health information that she’s got, there’s that, that, that. But you don’t have my name, you don’t have my address, you don’t have my phone number. Even if that goes to somebody else, all he knows is that number. He doesn’t know that it’s me. Even if my husband is conducting a research, he won’t still know that it’s his wife. So it doesn’t really matter.* (Mel)

*As long as information is being separated and it is not name orientated or where somebody lives. …. But, if there is no linkage to the data, then there’s no personal - it’s not really personal then, is it, if it’s not getting linked? If it’s just a statistic or a number it’s not really…* (Holly)

*I think the fact that there is no identifying the people involved. They could be somebody sitting next to them and they just don’t know. To them it’s just information to use for research; so no, not at all. I don’t think it’s an issue.* (Teresa)

Participants in this study who expressed the view that de-identified data is just ‘information’ did not support the no consent option indiscriminately. Rather, they sometimes chose the consent option, potentially indicating in this way that they were still in the process of assimilating new understandings with previously held views about consent.

### Consent choices across scenarios

The overall consent preferences for all scenarios are summarized in Table 3.

**Table 3:** Percentage of consent preferences across scenarios

	Consent should be sought	Consent need not be sought	Notification only
Scenario 1	35 % (*n* = 9)	58 % (*n* = 15)	8 % (*n* = 2)
Scenario 2	19 % (*n* = 5)	73 % (*n* = 19)	8 % (*n* = 2)
Scenario 3	15 % (*n* = 4)	85 % (*n* = 22)	0 % (*n* = 0)
Scenario 4	69 % (*n* = 18)	12 % (*n* = 3)	19 % (*n* = 5)

Participants who supported the ‘no consent’ option did not view the initial disclosure of personally identifying information for the purpose of creating the linkage key as a concern but some did indicate that the data linkage organization would need to be trustworthy. The focal point for participants was very much the analysis of the linked data rather than the handling of personal information in the initial data linkage stages. Concerns about identifiability arose prominently in Scenario 4 where the linkage was to be done by researchers, even though participants were assured that researchers would be required to de-identify data as soon as practicable. It was not so much a lack of trust in researchers that raised concerns, but, for some, the fear that a person known to them might access the data and discover things about them that they did not wish to reveal.

All participants supported the use of data in any form of research that would benefit society. Benefits arising from data linkage were also a major focus in responses regarding the non-consensual use of health data. During the discussions about the scenarios, participants indicated that they were positively disposed to participating in research. Despite their support for research, participants recognized that people do have a right to refrain from contributing but that they should nevertheless be able to enjoy the benefits arising from research.

### Key findings

There were two key findings arising in this research in relation to the final consent choices selected by the majority of the participants:

#### Key finding 1

As can be seen in Table *3*, the preferred majority consent option throughout scenarios 1–3 was ‘no consent’ following numerous shifts between choices, as discussed in the following section.

The no consent preference consistently increased from Scenario 1 through to Scenario 3. Scenarios 2 and 3, by far, showed the greatest support for the ‘no consent’ option. The point of note in relation to Scenario 3 is that, although it evoked a number of strongly-held views about why these hypothetical participants experiencing mental health issues should be given the choice to consent, a number of participants appeared to view their circumstances as a reason not to seek consent. The no consent option was further supported by the fact that the public benefits of this research were seen to be of considerable import given the potential risk of harm to members of the community from people such as the hypothetical participants of Scenario 3.

#### Key finding 2

Unlike Scenarios 1–3, Scenario 4 attracted a majority view that consent should be sought, as a result of the linkage being conducted by researchers rather than a dedicated linkage organisation. Scenario 4 also differed from the other scenarios in that it yielded the highest percentage of preferences for *notification of research*.

### Shifts between consent choices

There were multiple shifts in views regarding participants’ consent preferences for each scenario as shown in Column A of Table 4. There was a decrease in constant *consent* preferences across scenarios, except in Scenario 4 (row a), and an increase in constant *no consent* preferences across scenarios, with the exception of Scenario 4 where a constant view that consent should be sought was held by the majority of participants (*n* = 15). Switches from the consent to the no consent preference were far greater in Scenario 1 but persisted in some participants throughout the scenarios (row c). Given the nature of Scenario 4, it is perhaps unsurprising that this scenario involved the largest number of switches from the no consent to the consent preference (row e) or from an initial no consent preference to notification of research (rows i, j).

**Table 4:** Constant views and shifts in views within scenarios

(Column A)	Scenario 1	Scenario 2		Scenario 3	Scenario 4
Consent shifts and final consent decisions	No. of participants	No. of participants		No. of participants	No. of participants
		ND^a^	D^b^		
a. Consent	5	4	2	3	15 (8 = OI^c^, 7 = OO^d^)
b. No consent	4	17	22	21	1
c. Consent → No consent	11	3	2	1	1
d. Consent → No consent → Consent	3	1	--	--	--
e. No consent → Consent	1	--	--	1	4 (1 = OI/3 = OO)
f. Consent → No consent → Inform of research findings	1	--	--	--	--
g. No consent → Notification of research → Inform of research findings	1	--	--	--	--
h. Consent → No consent → Notification → Consent	--	1	--	--	--
i. No consent → Notification	--	--	--	--	1
j. No consent → Consent → Notification	--	--	--	--	3
k. No consent → Consent → Notification → No consent	--	--	--	--	1
Total participants	26	26	26	26	26

^a^ND = not deceased, ^b^D = deceased, ^c^OI = opt in, ^d^OO = opt out

### Justifications for consent choices – Individual scenario findings

The full range of justifications offered for the consent and no consent preferences expressed are shown in Tables 5, 6, 7, 8 below. Most participants offered more than one justification and sometimes provided different or additional justifications when reverting back to an original position or shifting to a new consent choice. The presentation of justifications in this paper aims to demonstrate the great diversity of views revealing the importance that lay people attach to consent in research or reasons why people think consent need not be sought. It is evident that many bear resemblance to each other in content yet these have been preserved as separate justifications so that the subtle nuances are not lost. The justifications have been further analyzed and will be presented elsewhere in greater detail.

**Table 5:** Scenario 1 justifications for consent choices

Scenario 1 - health data linked by experts	
Consent justifications	No consent justifications
People have right to choose (6)^a^	Use of de-identified data does not require consent (12)
People need to be aware of what is happening (6)	Acceptable practice because of the benefits (9)
To ensure people are not upset/do not object (6)	Large data sets serve as protection against identification (3)
Consent should be sought at initial point of data collection (5)	Strict measures/guidelines provide protection (3)
Information belongs to people (4)	Participants are not directly involved (3)
People prefer to be given the choice (3)	Practical considerations of obtaining consent from thousands (3)
Consent ensures that privacy is protected (3)	
Seeking consent is a sign of respect (2)	Consent lowers research participation rates (3)
Consent provides protection (2)	Knowledge of data linkage process allays concerns so no consent is acceptable (2)
Use of information without consent leads to trouble for researchers (2)	
Consent is required for everything (2)	Acceptable if security and safety measures in place (2)
Consent should be sought for all research (2)	Privacy legislation binds researchers and protects participants (2) The more participants involved, the better the quality of the study (1)
Disclosure of information to a third party requires consent (2)	No need for consent if data linkage organisation is trustworthy (1)
Unfair to force participation (1)	Medical information will not be provided to other parties (1)
People want to control their information (1)	
Some don’t trust that information stays anonymous (1)	Use of de-identified information does not breach privacy (1)
Seeking consent is courteous (1)	Retrospective use of data does not require consent (1)
Consent required because of cultural differences; some people don’t like their information used if they derive no benefits, or if there are perceived risks (1)	Acceptable depending on study (1)
Consent is required when researchers access identifiable information (1)	Acceptable due to cost and time constraints involved when obtaining consent (1)
Consent is required when other spheres of life (apart from health) are involved (1)	
Seeking consent for use of private information is an ethical requirement (1)	
Consent is a legal requirement (1)	
To cater for future uses of the same data (1)	

^a^Bracketed numbers reveal the number of participants offering each justification

**Table 6:** Scenario 2 justifications for consent choices

Scenario 2 - health data linked by experts	
Consent justifications	No consent justifications
Consent should be sought at initial point of data collection (4)^a^	Use of de-identified data does not require consent (13/D9^b^)
To ensure people are not upset/do not object (2)	Acceptable practice because of the benefits (8/D1)
People have right to choose (1)	Practical considerations of obtaining consent from thousands (4/D2)
Information belongs to people (1)	Audit type activities do not require consent (3/D1)
People want to control their information (1)	Acceptable due to cost and time constraints involved when obtaining consent (3/D2)
Consent for deceased person’s information should be sought because they still have rights (D1)	
Seeking consent is a sign of respect (1/D1)^b^	Requesting consent for use of de-identified data is burdensome, as people are time-poor (1/D1)
Seeking consent for deceased people’s information is appropriate because knowledge that they are contributing to society would bring comfort to families (D1)	Requesting consent could create difficulties, as people’s confidence in the fact that research uses de-identified data might be reduced (1)
	Acceptable if security and safety measures in place (1)
Use of information without consent leads to trouble for researchers (1)	Use of information will not prejudice participants (1)
Not seeking consent infringes privacy (1)	Acceptable not to seek consent from those who cannot be contacted if strict guidelines adhered to (1)
Consent should be sought for all research (1)	Not dealing with people directly (1)
Consent for use of data should be sought whenever health services are accessed. Therefore, a deceased person’s consent would have already been obtained (D1)	Retrospective use of medical data acceptable (1)
	Requesting consent for deceased people’s information could traumatise family (D5)
There are ways to achieve contact to request consent despite difficulties (1)	Data regarding deceased people is invaluable and should be included (D2)
	Relatives will be unaware of use of data relating to deceased relative (D2)
	Use of deceased people’s information does not impact on deceased person or their family (D2)

^a^Bracketed numbers reveal the number of participants offering each justification

^b^ *D* denotes justifications provided in relation to deceased individuals’ information

**Table 7:** Scenario 3 justifications for consent choices

Scenario 3 - health & criminal records data linked by experts	
Consent justifications	No consent justifications
Sensitivity of data requires that consent be obtained (2)^a^	Acceptable practice because of the benefits (15)
The more sensitive the data, the greater the need to obtain consent (2)	Use of de-identified data does not require consent (12)
	Practical considerations of obtaining consent from thousands (5)
People have right to choose (1)	Given the difficulties in obtaining consent, it is best to conduct research as benefits would be great (4)
Not seeking consent infringes privacy (1)	
Consent should be sought for all research (1)	Research focusing on issues such as violence, which affects others/the whole community, justifies not obtaining consent (3)
Mental health issues do not warrant not obtaining consent from participant or authorised carer (1)	Strict measures/guidelines provide protection (2)
The need for large number of participants is no excuse for not considering obtaining consent (1)	Acceptable to do mental health research without consent (2)
	Consent not very important where safety issues are concerned (2)
Research involving ‘vulnerable’ people requires consent (1)	When you weigh up individual vs. community benefits, community benefits here are greater (1)
If there is a legal guardian, consent should probably be sought (1)	No harm to individuals because they will not be named (or ‘outed’) (1)
	No impact on participants, who will be unaware that their data were used (1)
	Some participants cannot consent (1)
	Trying to get consent (including from relatives) could delay research, which
	should be done promptly because if its nature (1)
	Some participants may not have guardians (1)

^a^Bracketed numbers reveal the number of participants offering each justification

**Table 8:** Scenario 4 justifications for consent choices

Scenario 4 - health, Work Cover & employment data linked by researchers	
Consent justifications	No consent justifications
Consent provides protection against potential impact of research (10)^a^	Acceptable practice because of the benefits (5)
Access to identifiable data by researchers requires consent (9)	The findings are presented in de-identified form (3)
	Practical considerations of obtaining consent from thousands (3)
Consent required when researchers do the linkage (7)	Acceptable due to cost and time constraints involved when obtaining consent (2)
Consent is required when other spheres of life (apart from health) are involved (7)	Consent lowers research participation rates (2)
Opt-out consent would result in more participants being involved (6)	Low participation rates impact on quality of research (2)
Consent required because of lack of separation of tasks (4)	The information is already there so it should just be used without consent (2)
People have right to choose (4)	
Researchers may disclose information to third party (2)	Researchers are not interested in specific cases (2)
Someone on the research team may know the research participants (2)	Strict measures/guidelines provide protection (2)
To ensure people are not upset/do not object (2)	Getting consent could have detrimental effect on participants (1)
	No need for consent if data linkage organisation is trustworthy (1)
Opt-in consent captures the people who really want to participate (2)	Researchers will not use information obtained without consent to harm participants (1)
Having the option to consent is good (2)	
Information belongs to people (1)	Provided that the linkage organisation was involved so that tasks are separated (1)
People want to control their information (1)	
Researchers using identifiable information without consent is intrusive (1)	Researchers will be dealing with de-identified data eventually (1)
	Acceptable depending on study (1)
Consent required when participants are experiencing mental health issues, as they may not welcome people delving into their affairs at that point in their lives (1)	Consent would have been given at data collection point (1)
	Information given to WorkCover can be shared with researchers, as it was given confidentially (1)
Consent should be sought for all research (1)	Retrospective use of data does not require consent (1)
Consent should be sought at initial point of data collection (1)	People have inflated view of how interesting they are to others (1)
Collecting this kind of information without consent may not be legal (1)	
It is very good to obtain consent if it is simple to do so (1)	

^a^Bracketed numbers reveal the number of participants offering each justification

The justifications provided both for consent and no consent in Scenario 1 covered a range of areas pertaining to ethical, legal, and socially acceptable practices (Table 5). The justifications for consent revealed participants’ understanding of their rights as research participants, the functions that consent is generally perceived to hold (such as protection of privacy), the legal and ethical requirements surrounding consent as well as the social expectations surrounding consent. Participants’ justifications also provide insight into the importance people attach to being given the opportunity to make the choice for or against participation in research. Conversely, the justifications for the no consent option revealed that participants considered the protection mechanisms that data linkage provides in addition to protection mechanisms provided via guidelines and legislation and the nature of the research itself.

In Scenario 2 (Table 6) many of the justifications for the consent preference were the same or similar to the views expressed in Scenario 1. There was a marked decrease, however, both in the range and number of justifications for consent but some justifications related specifically to information about deceased individuals. For example, one participant felt that deceased individuals still have some ‘rights’ over their information while others felt that seeking consent is a display of respect for both individuals who are alive and those deceased. The no consent justifications, on the other hand, increased both in number and range with some also referring specifically to information about deceased individuals. The latter took into account both risks and the impact seeking consent might have on the family. Participants’ justifications for the no consent option revealed that they had some understanding of the different types of research activities and the practicalities involved. For example, some participants referred to audit type activities and felt that consent is not required for such activities. In addition, participants also seemed to be aware of the trade-off between non-consensual uses of data and benefits arising from research.

The majority of justifications for the consent option in Scenario 3 (Table 7) related to the sensitivity of the data being contemplated for use or the status of the research participants who were seen to require greater levels of protection via mechanisms such as consent. Some participants were aware that there are options for substituted decision-making when the person directly involved is unable to provide consent. The no consent justifications prominently supported two sets of views: that research employing non-identifiable data can proceed without consent because of the protections the data linkage process affords; and that research which may provide findings that could ultimately lead to better community protection should proceed without consent, as the benefits of such research were seen to outweigh the benefits of obtaining consent. Some participants displayed a lack of appreciation of protections that should be afforded to all research participants irrespective of their health or criminal status.

More than half the justifications for the consent preference in Scenario 4 (Table 8) related directly to the fact that researchers would be undertaking the linkage themselves and the perceived harms arising from researcher involvement in this process. Other justifications indicated participants’ discomfort with the nature of the information being linked and there were suggestions about the kind of consent that could be sought (i.e. opt-in or opt-out consent).

The justifications for the no consent option were half in number compared to the justifications for the consent option. Even though the vast majority of participants supported the consent option for this scenario, some participants started out supporting the no consent option and gave justifications but then reverted to the consent option or the notification option. The greatest number of justifications for no consent related to the impact trying to obtain consent would have on the research but others spoke of the eventual use of de-identified data, the benefits arising from the research and the fact that researchers are not interested in specific individuals’ data. Despite clarifications that no data linkage organization would be involved, some participants insisted that such research would be acceptable without consent only if a trustworthy data linkage organization were involved. This highlights their non-acceptance of researchers themselves undertaking the linkage.

### Non-consensual use of health data

Following the discussion on the four hypothetical scenarios, participants were asked if it was ever acceptable to use health data without obtaining consent. The majority (*n* = 18) indicated that it was acceptable and the main justification themes that arose related to anonymity and safety measures (*n* = 17), benefits that arise from data linkage research (*n* = 14), and the reduction of harm to participants offered via data linkage processes (*n* = 6). A large number of additional justifications was provided but these were expressed by few participants. Some examples include: wastage of resources required to obtain consent from large numbers (*n* = 2), an understanding of data linkage process promotes agreement for research to be conducted without consent (*n* = 2), and that the ability to conduct research is ‘more important than one individual’s right to give consent or not’ (*n* = 1).

Fewer participants (*n* = 7) indicated that non-consensual use of health data was never acceptable, with three of these participants changing their view in favour of non-consensual use of such data to supporting the consent option. The main justification related to the need for people to be able to make the choice regarding participation themselves (*n* = 4) followed by the belief that secondary uses of data require consent (*n* = 2).

## Discussion

### Consideration of pre-research assumptions

The pre-research assumption that participants will generally have a limited understanding of what data linkage is and what it involves proved to hold. As a result, an explanation of data linkage processes was required during the interview, which proved extremely easy for participants to understand. This suggests that lay people with little or no recollection or understanding of the complex processes data linkage involves are able to quickly grasp the basic concepts involved in order to discuss pertinent ethical and social issues.

The second assumption that participants would prefer to opt in to data linkage projects did not hold, as most participants considered it appropriate to conduct data linkage projects without consent provided there is separation of tasks so that researchers do not obtain identifiable data. The reasons they cited as influencing this position included the protections provided and the de-identifiable data used. This preference is also reflected in findings from an Irish study where the vast majority of participants were comfortable with GPs releasing de-identified data for research purposes [8] as well as findings from a systematic review of studies reporting on consent proportions to record linkage [27]. Similar views were expressed in research conducted in the UK but some participants in this research continued to view de-identified data as belonging to them because it was about them [28].

Finally, in contrast to the assumption that most will prefer to opt in irrespective of identifiability, respondents supported the non-consensual use of data and appeared to make a distinction between the use of identifiable and non-identifiable data. This finding conflicts with views expressed by Australians in a nation-wide study conducted by King and colleagues [7]. In that study, 92 % of survey participants expressed the view that consent should be sought for the use of their health information when used for purposes other than treatment. The disparity between the findings may be explained by the fact that participants in the King et al. study [7] were considering research conducted with electronic health records and were advised that de-identified data can be linked back to identifying information. In the current study, however, participants were informed that the data linkage process considered and the strict regulations surrounding such uses of data did not permit de-identified data to be linked back to identifying data.

### Consideration of key findings

Key finding 1 was that the preferred majority consent option throughout scenarios 1–3 was ‘no consent’. There were, however, variations in preferences throughout the scenarios. An explanation of the variations throughout the scenarios (Table 3) could be that, in Scenario 1, participants had just had the concepts (i.e. data linkage) explained to them, and by admission from a number of participants, their initial spontaneous consent preference had arisen from previously strongly-held views, which did not necessarily apply in the case of data linkage given the protective measures in place to preserve privacy. The fact that the no consent option increased progressively from Scenario 1 through to Scenario 3 may indicate that participants were becoming more accepting of the notion of no consent in the context of data linkage as a result of an understanding of the processes and protections involved. Similarly, they may have been relinquishing previously held automated responses supporting the consent option, resulting from the very high value our society places on individual rights but also their lack of understanding of data linkage processes.

Key finding 2 was that Scenario 4 attracted a majority view that consent should be sought and yielded the highest percentage of preferences for notification of research. The reverse order of preferences in Scenario 4 was a function of the multiple disparate datasets that were to be accessed and the fact that there was no separation of tasks, i.e. the researchers would be accessing identifiable data to create the linkage key and also doing the analysis. The majority of participants were concerned that researchers would be accessing identifiable data and the fact that they insisted on consent being sought in this Scenario indicates that they not only understood the function and protections afforded by the linkage organization, but also, that they were not indiscriminately choosing the consent option without due consideration of the facts of each scenario. By this stage, some had even adopted the language used by experts, that is, *separation of tasks*, even though this expression was not used throughout the discussion but only arose in the explanation provided at the start of the scenarios.

Increased support for the ‘notification only’ option may have arisen as a compromise by those who would have preferred the ‘no consent’ option but were also conscious of the sensitivity of the data being linked. Participants had concerns that linkage of such (identifiable) data enables the creation of a much more comprehensive picture of the individual.

### Complexity of consent choices

The design of the study enabled the identification of participants’ consent preferences (consent, no consent, notification), including the oftentimes multiple shifts in views, before settling on their final choice. It also revealed participant justifications for choices made. Therefore, it enabled the tracking of participant views both within and across the scenarios thus highlighting the complexity of such consent decisions. Participants considered concrete scenarios relating exclusively to data linkage; this necessarily emphasized the processes involved in data linkage, the potential real or perceived risks of harm, and the potential benefits arising from such research. It also necessarily brought into focus the constraints that stringent consent requirements impose on the conduct of data linkage projects. Directing participants’ focus towards these issues throughout the interview enabled a better processing of the issues that require consideration, rather than having participants disperse their attention on numerous contextually different uses of personal and health information, which may involve a slightly different set of ethical and social considerations.

Even without considering the reasons why participants switched views, these switches provide a clear indication of the multiple considerations participants had in relation to preferred consent choices when the opportunity to consider pertinent related issues was provided and when there are no constraints on time or space to alter, perhaps deeply ingrained, initial preferences.

At the outset, participants were assumed to know very little about data linkage despite their previous involvement in the related RCT. Given the complexity of best practice data linkage processes discussed with participants, an explanation of data linkage processes was given with the aid of a diagram outlining the key processes. Despite some protestations from participants regarding the complexity of the concepts, participants demonstrated that they were able to consider these carefully and they articulated their views in varied and interesting ways.

### Support for data linkage

Participants in this study generally supported research and this finding is consistent with that of other studies that show that people generally support research in both Australian [7] and international contexts [14, 16]. However, in contrast to the findings of this study, in the aforementioned studies people’s general support of research did not align with a preference for no consent. People in these studies still wished to be informed of the use to which their data would be put.

Overall, participants were very supportive of data linkage research but did hold concerns regarding the linkage of certain kinds of data, especially data related to employment. It was not clear whether their concerns were about the use of employment data or the fact that in the scenario presented the linkage was to be done by researchers. Participants in a Canadian study were also more reluctant to have information about income, occupation and education linked to health data, with 30–40 % of individuals both from the general public and with a specific health condition expressing the need for consent in such a scenario [11]. Conversely, the same participants were more accepting of the linkage of health data and biological samples (with no commercial profit) [11]. Participants in this study were most comfortable with the linkage of health data but were willing for other kinds of data to be linked if the public benefits arising from the research were deemed to be great. This especially applied to research which directed its focus to an understanding of violence against others (Scenario 3), which they assumed would ultimately translate into a reduction of such behaviours. Participants’ support for data linkage was further confirmed when non-consensual use of health data was discussed more broadly and not specifically relating to the scenarios.

Most participants were not concerned about the initial necessary use of identifiable data to create the linkage key. This finding may point to one of two things: that views regarding privacy are changing, or that the restrictions placed on health and personal data have not reflected community views but rather have reflected concerns by legislators and administrators. The focal point for participants was very much the analysis of the linked data rather than the handling of personal information in the initial data linkage stages.

Concerns about researchers conducting the linkage themselves, which arose in scenario 4, reflected the views of Australians in a previous study. In the study by King et al. [7], 37 % of respondents (*n* = 700) would be concerned or very concerned if the use of the de-identified health information could lead to them being identified, 29 % were slightly concerned while 33 % indicated that they would not be concerned about the use of their de-identified health information even if it could be traced back to their identity.

### Balancing conflicting values

The findings indicate that participants had a wealth of understanding and knowledge, which they themselves had been unaware of and which several of them had underestimated, as made apparent by their view that they had never thought about these issues before and were therefore not knowledgeable enough to comment. Participants demonstrated an understanding of the need to balance public benefits with the protection of privacy. A noteworthy feature was the struggle that participants experienced when trying to settle on their final position. This was due to conflicting values, notably between privacy protection and public benefits, which participants had to consider throughout the scenarios. Many participants made numerous switches within each scenario with the thought process, when vocalized, resembling that of a debate. Some participants were concerned that they would seem inconsistent if they made different choices for different scenarios. This may indicate that we are relatively inflexible with regard to issues such as consent and not readily accustomed to weighing up opposing values. In fact, the spontaneous response regarding the need for consent, especially during the first scenario, was confessed by some to have been offered out of habit, as they perceived that most things require consent: “*I know that you need to get consent for everything*” (Henrietta) or “*I mean instantly I thought, yes, they should be asked* [for consent] *but really there’s no impact on them”* (Margaret). This may be indicative of a culture which places greater value on the individual rather than the community as a whole. The swing to the consent option and the justifications provided in Scenario 4 indicated great unease when an independent organization is not involved in creating the linkage key and therefore researchers need to deal with this aspect before they can analyze de-identified data.

The justifications provided both in support of consent and no consent, as well as notification, where discussed, were rich and diverse. Those who supported the consent option throughout did not seem to provide justifications of a different nature compared to those who changed views depending on the specifics of the scenario, as the latter also raised issues of rights, protections provided by consent, the need to control information, and consent being a mark of respect towards people.

### Practical implications of consent requirements in data linkage

A number of participants were cognizant of the practical implications of seeking consent from large cohorts, as were the majority of participants in a study examining a Scottish data linkage system [10]. However, many participants in the current study only considered this aspect as well as the implications for selection bias when the issue was raised by the researcher. A lack of understanding of such details may be commonplace given that research is not widely discussed in the media or in public discourse. This is an issue which impacts on the public’s ability to arrive at informed views about data linkage. Research participants in another study were also more positively disposed to non-consensual use of data when they better understood the impact of stringent consent requirements [29].

Data linkage processes have further developed over the past few decades and they provide a high level of protection for people whose data are used. The value of such activities is now greatly recognized but involvement of the broader public in discussions regarding the appropriate consent processes has lagged. Findings such as these should encourage greater discussion with the public, who are entitled to be provided with an understanding of how their data may be used. Even if some disagree with the non-consensual use of de-identified data, it may be morally permissible to use such data for the greater societal good. However, we place ourselves in a morally perilous position if we are not open and transparent about how we use people’s data, even if we engage in such activities with the aim of providing broader social benefits.

### Limitations of the research

This research presents what may appear to be constraints typical of qualitative research; that of numbers and generalizability to larger populations. It must be borne in mind, however, that the purpose of qualitative research is not to be able to generalize to populations but to be able to make generalizations regarding the phenomenon under consideration [30], which in this paper relates to lay views on consent and the acceptability of non-consensual use of health data in data linkage.

It has been shown that consenting participants share characteristics different to those of non-consenters, a fact which can bias research findings [31–33] and can therefore have consequences for evidence-based policies or processes that arise from the research. Even though people willing to participate in research often differ from those not willing to do so, the reasons for non-participation in this research are believed to primarily relate to family and personal circumstances or inability to contact potential participants.

### Future research

This research revealed that participants did not engage with the concept of notification of future use of data at the initial point of collection of information, e.g. hospital. This issue warrants further investigation, as this process appears to provide both a respectful and informative solution to individuals whose data may be used in data linkage projects. Since the research, there have been significant breaches in privacy world-wide and it is therefore necessary to examine whether the greater risks we are more generally apparently exposed to impacts on people’s perception of consent requirements in data linkage projects.

## Conclusion

These lay participants showed a good understanding of issues surrounding consent and its application and relevance to data linkage. The views expressed indicate that lay people have the ability to discern issues of philosophical, cultural, and political relevance and gain an understanding of pertinent issues within a relatively short exchange and limited exposure to the complex nature of data linkage.

Overall, there was support for the no consent option, when protections were deemed to be adequate, especially, for example, if researchers did not access identifiable data. Many participants thought that health information that was not linked to specific individuals any longer did not hold the same value and could be used for research purposes without consent. The majority of these participants did not appear to think that notification of future use of data was necessary. This finding does not require the abandonment of this method of involving the public but does certainly enrich our understanding of the views held by some members of the public.

The multiple shifts between consent preferences were evidence that lay people can and do consider the conflicting values and interests that arise when considerations central to the protection of people’s information, societal benefits, and the nature and constraints of research need to be balanced. Finally, this research indicates that the public can quite readily understand enough about data linkage to discern between uses of data that might cause harm and uses that include adequate protections.

It is incumbent upon governments engaged in the development of data linkage infrastructure and researchers who apply these research methods to more broadly publicize both the methods adopted in data linkage and the benefits arising from such activities.

## Footnotes

1 Of note is the fact that the linkage proposed in this study involved linking health information to work, education, or income data, the nature of which may have resulted in the strong preference for consent.

## References

[1] Holman CD, Bass AJ, Rosman DL, Smith MB, Semmens JB, Glasson EJ, et al. A decade of data linkage in Western Australia: strategic design, applications and benefits of the WA data linkage system. Aust Health Rev. 2008;32(4):766–77. 10.1071/ah080766 18980573

[2] Brook EL, Rosman DL, Holman CD (2008). Public good through data linkage: measuring research outputs from the Western Australian Data Linkage System. Aust N Z J Public Health.

[3] Barry SJE, Dinnett E, Kean S, Gaw A, Ford I (2013). Are Routinely Collected NHS Administrative Records Suitable for Endpoint Identification in Clinical Trials? Evidence from the West of Scotland Coronary Prevention Study. PLoS ONE.

[4] Hall SE, D’Arcy C, Holman J, Finn J, Semmens JB (2005). Improving the evidence base for promoting quality and equity of surgical care using population-based linkage of administrative health records. Int J Qual Health Care.

[5] Population Health Research Network. Available from: http://www.phrn.org.au [accessed 11 April 2011].

[6] Berry JG, Ryan P, Duszynski KM, Braunack-Mayer AJ, Carlson J, Xafis V, et al. Parent perspectives on consent for the linkage of data to evaluate vaccine safety: A randomised trial of opt-in and opt-out consent. Clin Trials. 2013;10(3):483–94. 10.1177/1740774513480568 23568940

[7] King T, Brankovic L, Gillard P (2012). Perspectives of Australian adults about protecting the privacy of their health information in statistical databases. Int J Med Inform.

[8] Buckley BS, Murphy AW, MacFarlane AE (2011). Public attitudes to the use in research of personal health information from general practitioners’ records: a survey of the Irish general public. J Med Ethics.

[9] Perera G, Holbrook A, Thabane L, Foster G, Willison DJ, et al. Views on health information sharing and privacy from primary care practices using electronic medical records. Int J Med Inform. 2011;80:94–101. 10.1016/j.ijmedinf.2010.11.005 21167771

[10] Haddow G, Bruce A, Sathanandam S, Wyatt JC (2011). ‘Nothing is really safe’: a focus group study on the process of anonymizing and sharing of health data for research purposes. J of Eval in Clin Prac.

[11] Willison DJ, Steeves V, Charles C, Schwartz L, Ranford J, Agarwal G (2009). Consent for use of personal information for health research: do people with potentially stigmatizing health conditions and the general public differ in their opinions?. BMC Med Ethics.

[12] Willison DJ, Swinton M, Schwartz L, Abelson J, Charles C, Northrup D (2008). Alternatives to project-specific consent for access to personal information for health research: insights from a public dialogue. BMC Med Ethics.

[13] Campbell B, Thomson H, Slater J, Coward C, Wyatt K, Sweeney K (2007). Extracting information from hospital records: what patients think about consent. Qual Saf Health Care.

[14] Damschroder LJ, Pritts JL, Neblo MA, Kalarickal RJ, Creswell JW, Hayward RA (2007). Patients, privacy and trust: Patients’ willingness to allow researchers to access their medical records. Soc Sci Med.

[15] Williams B, Irvine L, McGinnis AR, McMurdo ME, Crombie IK (2007). When “no” might not quite mean “no”; the importance of informed and meaningful non-consent: results from a survey of individuals refusing participation in a health-related research project. BMC Health Serv Res.

[16] Willison DJ, Keshavjee K, Nair K, Goldsmith C, Holbrook AM (2003). Patients’ consent preferences for research uses of information in electronic medical records: interview and survey data. BMJ.

[17] Barrett G, Cassell JA, Peacock JL, Coleman MP (2006). National survey of British public’s views on use of identifiable medical data by the National Cancer Registry. BMJ.

[18] Berry JG, Ryan P, Braunack-Mayer AJ, Duszynski KM, Xafis V, Gold MS (2011). A randomised controlled trial to compare opt-in and opt-out parental consent for childhood vaccine safety surveillance using data linkage: study protocol. Trials.

[19] King G, Heaney DJ, Boddy D, O'Donnell CA, Clark JS, Mair FS (2011). Exploring public perspectives on e-health: Findings from two citizen juries. Health Expect.

[20] Ipsos MORI (2007). The Use of Personal Health Information in Medical Research General Public Consultation.

[21] Kass NE, Natowicz MR, Hull SC, Faden RR, Plantinga L, Gostin LO (2003). The use of medical records in research: What do patients want?. J. Law Med. Ethics.

[22] Laws PJ, Hilder L (2008). Australia’s mothers and babies 2006.

[23] Ritchie J, Spencer L, Bryman A, Burgess RG (1994). Qualitative data analysis for applied policy research. Analyzing Qualitative Data.

[24] Pope C, Ziebland S, Mays N (2000). Qualitative research in health care: Analysing qualitative data. BMJ.

[25] Ritchie J, Lewis J (2003). Qualitative research practice : a guide for social science students and researchers.

[26] NVivo qualitative data analysis software. 2008, QSR International Pty Ltd.

[27] da Silva MEM, Coeli CM, Ventura M, Palacios M, Magnanini MMF, Camargo TMCR, et al. Informed consent for record linkage: A systematic review. J Med Ethics. 2012;38:639–42. 10.1136/medethics-2011-100208 22403083

[28] Castell S, Charlton A, Clemence M, Pettigrew N, Pope S, Quigley A, et al. Public Attitudes to Science 2014: Main Report. 2014, Ipsos MORI (retrieved at: https://www.ipsos-mori.com/Assets/Docs/Polls/pas-2014-main-report.pdf November 2014).

[29] Hill EM, Turner EL, Martin RM, Donovan JL (2013). “Let’s get the best quality research we can”: Public awareness and acceptance of consent to use existing data in health research: A systematic review and qualitative study. BMC Med Res Methodol.

[30] Liamputtong P, Ezzy D (2005). Qualitative research methods.

[31] Woolf S, Rothemich S, Johnson R, Marsland D (2000). Selection bias from requiring patients to give consent to examine data for health services research. Arch Family Med.

[32] Kho M, Duffett M, Willison D, Cook D, Brouwers M (2009). Written informed consent and selection bias in observational studies using medical records: systematic review. Br Med J.

[33] Damery S, Ryan R, McManus RJ, Warmington S, Draper H, Wilson S. The effect of seeking consent on the representativeness of patient cohorts: iron-deficiency anaemia and colorectal cancer. 2011.doi:10.1111/j.1463-1318.2011.02724.x 10.1111/j.1463-1318.2011.02724.x 21831101

